# Spontaneous Colovesical Fistula With Benign Prostatic Hyperplasia and Prostatitis

**DOI:** 10.7759/cureus.69589

**Published:** 2024-09-17

**Authors:** Karthik Raja Ravichandran, Ray Munguia-Vazquez, Ruban Nirmalan

**Affiliations:** 1 General Surgery, Indiana University School of Medicine, Indianapolis, USA; 2 Otolaryngology and Head and Neck Surgery, Indiana University School of Medicine, Indianapolis, USA

**Keywords:** colovesical fistula, laporoscopic, large bowel diverticula, peritonitis, prostatitis

## Abstract

We present an unusual case of colovesical fistula formed by a single diverticulum manifesting as prostatitis and urinary tract infection (UTI) before causing further complications. Diverticulitis is caused by inflammation of the colonic diverticula and manifests as fever and left lower quadrant pain. The patient also developed recurrent pneumaturia; upon examination, a colovesical fistula was observed on computed tomography (CT), which was managed successfully by laparoscopic repair. This case highlights manifestations of colovesical fistula secondary to diverticular disease and the laparoscopic approach to treatment.

## Introduction

Colovesical fistulas are commonly caused by diverticular disease with clinical manifestations including pneumaturia, fecaluria, suprapubic pain, and dysuria. It is more common in males, with an average age of presentation of 60. Diagnostic tools include computed tomography (CT) with contrast, urinalysis, and colonoscopy [1]. Colovesical fistulas are treated laparoscopically but have high morbidity and can lead to sepsis if not managed properly. Diverticular disease is common in Western countries due to a combination of genetics and diet, with the prevalence of colovesical fistulas ranging from 2% to 23% [2]. Prostatitis is also not a commonly reported complication in colovesical fistulas in the literature, with only one similar case reported [3]. Our patient was also younger than the average age of colovesical fistula patients. The presence of colonic diverticulosis in the population is about 40% and is more likely in older males. When diverticula get inflamed, they become symptomatic, causing fever, pain, and changes in bowel habits. Uncomplicated diverticulitis can be treated with bowel rest, analgesics, and broad-spectrum antibiotics, while complicated diverticulitis requires surgery due to the risk of perforation, which can cause peritonitis, sepsis, and death.

## Case presentation

A 43-year-old male presented to outpatient urology with dysuria, urgency, pneumaturia, and groin pain with a pertinent past medical history of benign prostatic hyperplasia. Family history is significant for colorectal cancer in his father. Urine culture showed a proteus growth sensitive to ciprofloxacin. Ciprofloxacin 500 mg twice a day was started for three weeks; however, symptoms returned, which prompted a course of trimethoprim/sulfamethoxazole 160 mg twice a day for 10 days, which resolved symptoms until pneumaturia recurred five months later. The patient presented to the emergency department with peritonitis in his right lower quadrant four months later. Upon examination, the patient was septic, and labs showed leukocytosis. Piperacillin/tazobactam and lactated ringers were started.

**Figure 1:**
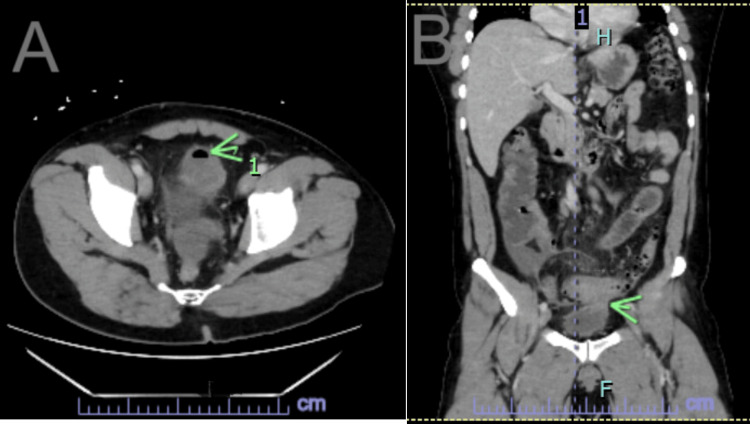
CT of colovesical fistula (A) Transverse; (B) Coronal

A CT scan of the abdomen and pelvis was done with 100 mL of Isovue-370 upon presentation to the emergency room, which showed long segment wall thickening and inflammatory stranding of the mid-sigmoid colon consistent with diverticulitis. Intraluminal air in the anterior bladder and wall thickening of the dome of the bladder raised suspicion of colovesical fistula (Figures 1A, 1B). Free fluid was present in the right lower quadrant, anterior pelvis, and the rectovesical pouch. Once the abscess was laparoscopically drained, CT showed resolution but residual stranding of the midcolon with air, as shown by the arrow in Figure 1A. Fistulization between the colon and bladder is shown by the arrow in Figure 1B. The patient was scheduled for a colonoscopy to rule out colon cancer prior to proceeding with sigmoid colectomy and colovesical fistula repair.

**Figure 2:**
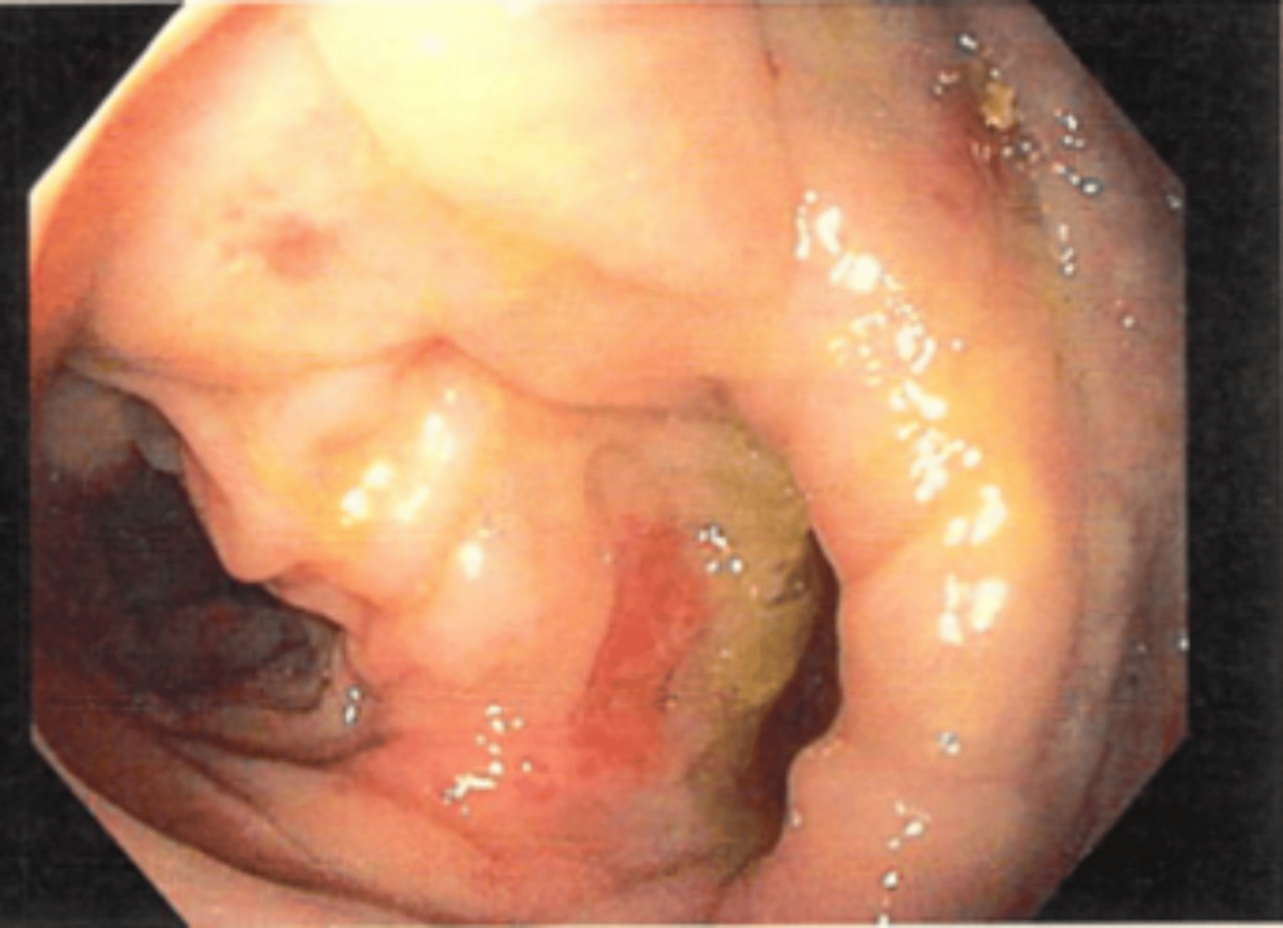
Diverticulum at sigmoid colon

Colonoscopy showed moderate diverticulosis in the sigmoid and descending colon with non-bleeding internal hemorrhoids (Figure 2). The patient met with urology prior to surgery for cystoscopy; findings included mucosal inflammation of the dome and enlarged prostate. The patient was able to proceed with surgery. In this case, colovesical fistula was the most likely diagnosis due to CT findings. However, mild dilation of the appendix was also observed. Leukocytosis, fever, abdominal pain, and nausea fit the picture of appendicitis. However, appendicitis would not explain the urinary tract infection (UTI) and one-year history of pneumaturia. The dilation is thought to be reactive due to the fistulization and pelvic fluid.

A Da Vinci-assisted laparoscopic sigmoid colectomy with colovesicular fistula repair was done. This is ideal for the patient due to faster recovery times and lower risk of infection. A preoperative epidural was administered after intubation, and the patient was placed in the lithotomy position. A transverse right periumbilical incision was made and subcutaneous tissue was dissected; the fascia was grasped with a coker while a Veress needle was passed and insufflated with CO_2_ to 15 mmHg. An 8 mm da Vinci port was inserted through the incision, followed by an 8 mm da Vinci laparoscope, which revealed film-like enteroenteric adhesions consistent with the diagnosis of a fistula. A 12 mm right lower quadrant, two 8 mm left upper quadrant ports, and a 5 mm right upper quadrant air seal port were placed under direct visualization. The robot was docked to the patient with a hook cautery in arm one, fenestrated bipolar grasper in arm three, and bowel grasper in arm four.

A medial to lateral dissection was started with the inferior mesenteric artery and vein skeletonized and sealed. Dissection was continued toward the rectum and presacral plane, then scissors were used to dissect the sigmoid colon off the bladder using minimal cautery and mostly sharp dissection. Then 350 cc of methylene blue was instilled in saline with no leak. A lateral to medial dissection was done up the white line of toldt to the splenic flexure, resulting in circumferential mobilization of the left colon, sigmoid colon, and upper rectum. The mesentery of the rectosigmoid junction was transected using an ENSEAL device, and the junction was transected using a 45-blue stapler. The mesentery of the sigmoid was transected up to the proximal sigmoid colon. An enterotomy was made in the bowel with a 25-mm anvil and scissor; the anvil was passed and brought to the sidewall to close the defect. This was repeated in the rectum. The staples were loosened, and the leak test showed no evidence of leakage, so the abdomen was irrigated, and the procedure finished with a dressing of incisions.

The patient was discharged a day later, educated on managing the Foley catheter, and prescribed acetaminophen for headaches. The patient had a Foley catheter removed a week later and reported no pneumaturia, pain, or fecaluria and had normal bowel movements. The patient was advised to refrain from strenuous activity for four weeks and to follow up as needed.

## Discussion

The patient was diagnosed and treated for benign prostatic hyperplasia and prostatitis a year before presentation. We believe this was the first manifestation of the colovesical fistula and that prostatitis was due to intestinal content passing through the fistula. The diagnosis of colovesical fistula is a clinical one since not many conditions involve pneumaturia outside of UTIs. However, CT with contrast is the gold standard for detecting colovesical fistula, followed by cystoscopy and barium enema (which can detect strictures) [4]. There is also the so-called poppy seed test in resource-constrained environments where a patient ingests 35-250 grams of poppy seeds, and due to the fistula, they will present in the urine undigested. While this method can confirm the existence of a fistula, it cannot provide details on location or rule out malignancy like imaging can.

Outside of diverticular disease, colovesical fistulas can be caused by neoplasms, inflammatory bowel disease, sepsis, radiation, trauma, or iatrogenic. The enterovesical fistula can be managed surgically or non-surgically, depending on the etiology. Non-surgical management involves bladder drainage, antibiotics, and total parenteral nutrition (TPN). This may work in patients who present with minimal symptoms or in those who may not be fit for surgery. For example, a patient with Crohn’s disease could develop complications if a laparotomy is done, so in this situation, a conservative option with prednisone can be tried. However, non-surgical management has limited success, which is why the surgical method is most common. Indications for surgery include trauma and sepsis. Most patients only require a one-stage procedure in which the fistula is removed and involved organs are closed and reanastomosed [5]. Postoperative care involves managing a Foley catheter for one to two weeks and consultations with a urologist and gastroenterologist for imaging and antibiotics as needed. Patient outcomes for enterovesical fistulas are good, but prognosis can be poor in those with comorbidities.

Something interesting in the literature is that although this case report features a laparoscopic procedure, there is still debate in terms of whether an open or minimally invasive procedure should be the gold standard, especially in more complicated diverticular disease [6]. This is due to a lack of high-quality data such as randomized control trials, a small sample size, and short follow-up. In most surgeries, laparoscopic would be preferred due to quicker recovery, fewer postoperative complications, and shorter hospital stays. In colovesical fistula repair, it is not uncommon for a laparoscopic procedure to convert to open. There is some heterogeneity in the literature; for example, Giovanni et al. claimed that open would be better due to high conversion rates and morbidity [7]. They do mention that laparoscopic surgery would be more feasible in cases with less inflammation. A retrospective analysis by Rizzuto et al. showed comparable conversion, mortality, and morbidity between open and laparoscopic methods [8]. When looking at this case, there was a single diverticula favoring the laparoscopic approach.

There are also other less common gastrointestinal fistulas, including pyeloenteral and prostatorectal fistulas. For example, prostatorectal fistulas can be caused by radical prostatectomy in the setting of prostate cancer. It can present similarly to colovesical fistula with urinary infection and fecaluria. Management includes surgical treatment or bowel diversion via catheters. Pyeloenteral fistulas can be secondary to penetrating trauma, complex kidney stones, or ingested foreign bodies. Management with this type of fistula includes drainage and bowel rest or nephrectomy if the kidney is non-functioning.

## Conclusions

Gastrointestinal fistulas can occur between epithelium-lined parts of the digestive system, resulting in abnormalities in flow, with colovesical fistulas being the most common. A thorough workup is necessary when suspicion of fistula is high. This case was unique due to the fact that a single diverticulum led to fistulization, with classic manifestations such as pneumaturia occurring later. Presentation can vary based on the parts involved, but surgery or conservative management is important to prevent further complications such as sepsis. Our case adds to the literature showing prostatitis related to fistulization and can inform urologists about an atypical manifestation of colovesical fistula.
